# Tryptophan residue of plasmid-encoded Pgp3 is important for *Chlamydia muridarum* to induce hydrosalpinx in mice

**DOI:** 10.3389/fmicb.2023.1216372

**Published:** 2023-07-11

**Authors:** Yumeng Huang, Haoqing Wu, Yina Sun, Yuanjun Liu

**Affiliations:** ^1^Tianjin Medical University General Hospital, Tianjin, China; ^2^National Health Commission (NHC) Key Laboratory of Hormones and Development, Tianjin Key Laboratory of Metabolic Diseases, Chu Hsien-I Memorial Hospital, Tianjin Institute of Endocrinology, Tianjin Medical University, Tianjin, China

**Keywords:** Pgp3, point mutation, *Chlamydia muridarum*, hydrosalpinx, chlamydial survival

## Abstract

The crucial role of plasmid-encoded protein Pgp3 in *Chlamydia* pathogenesis has been demonstrated in various animal models. Previous studies have revealed that the Pgp3-deficient *C. muridarum* mutant fails to induce hydrosalpinx after vaginal inoculation in mice. Structural analysis of *C. trachomatis* Pgp3 trimer has indicated that Trp234 may play a critical role in trimeric crystal packing interactions and that Tyr197 is involved at predominant cation-binding sites. In this study, we constructed *C. muridarum* transformants harboring Pgp3, Trp234, or Tyr197 point mutations (Pgp3W234A and Pgp3Y197A). C3H/HeJ mice infected with Pgp3W234A mutant failed to induce severe hydrosalpinx in the oviduct tissue, which largely phenocopied the full-length Pgp3-deficient *C. muridarum*. The Pgp3Y197A variant induced an intermediate severity of pathology. The attenuated pathogenicity caused by the Pgp3W234A mutant may be due to its decreased survival in the lower genital tracts of mice, reduced ascension to the oviduct, and milder induction of inflammatory cell infiltration in the oviduct tissue. Thus, our results point to an important amino acid residue involved in Pgp3 virulence, providing a potential therapeutic target for chlamydial infection.

## Introduction

*Chlamydia trachomatis* (CT) is an obligate intracellular bacterial pathogen that causes trachoma and sexually transmitted infections, afflicting millions of people globally. If untreated, it may result in blindness and infertility (Ault et al., 1998; Mishori et al., 2012). The mechanisms by which *C. trachomatis* induces pathology in the oviduct are poorly understood, and characterizing its virulence factors can be difficult because *C. trachomatis* induces only mild pathology in mouse models (Carmichael et al., 2013). *Chlamydia muridarum* (CM) is widely used to investigate CT pathogenesis because it can infect the genital tracts of mice and induce pathologies in the oviduct that closely resemble those observed in CT-infected women (De Clercq et al., 2013).

*Chlamydia* species harbor highly conserved 7.5 kb plasmid-encoding plasmid gene proteins 1 (Pgp1) through plasmid gene protein 8 (Pgp8). The plasmid acts as a central virulence factor in the pathogenesis of infection because plasmid-free *Chlamydia* is dramatically attenuated in both the genital tracts of mice and the ocular tissues of non-human primates (O'Connell et al., 2007; Carlson et al., 2008; Liu et al., 2014a; Zhong, 2017; Huang et al., 2019). Among these plasmid-encoded genes, Pgp1, Pgp2, Pgp6, and Pgp8 are essential for plasmid maintenance. Pgp4 regulates plasmid-encoded and chromosome genes, including GlgA, CT049-CT050, and CT142-CT144 (Gong et al., 2013; Song et al., 2013; Zhang et al., 2020). Although Pgp5 is a negative regulator of Pgp4-regulated genes, it significantly contributes to the induction of hydrosalpinx (Huang et al., 2015). Importantly, mouse studies have proven that plasmid-encoded Pgp3 is the critical virulence factor that is largely responsible for the pathological phenotype caused by the plasmid (Liu et al., 2014a; Zhong, 2021). Pgp3 has been found to be essential for establishing persistent infection (Yang et al., 2020). In previous studies that performed intravaginal inoculation of Pgp3-deficient CM organisms, we found that pathogenicity was attenuated, likely due to the reduced ascension, survival, and induction of inflammatory infiltration in the oviduct by the CM (Liu et al., 2014a).

Pgp3 is a trimeric protein that is secreted into host cell cytosol and is also associated with the outer membranes of bacteria. Secreted Pgp3 promotes persistent survival of *Chlamydia* possibly *via* fighting against host innate immunity effectors such as the cathelicidin-related antimicrobial peptide in mice or LL-37 in humans (Hou et al., 2015). Pgp3 trimer includes a C-terminal trimerization domain (Pgp3c) containing the tumor necrosis factor (TNF)-like domain, a triple helices middle domain (Pgp3m), and an N-terminal domain (Pgp3n) with a series of structural motifs commonly found in trimeric viral proteins. A previous study demonstrated that these Pgp3 domains must maintain their structural integrity to confer virulence (Huang et al., 2019). The Pgp3 predominant cation-binding site is a potassium ion in octahedral coordination involving each O atom of the three tyrosine side chains Tyr197 and three bound water-molecule O atoms. The potassium ion is positioned at the entrance of a solvent-filled channel that penetrates the trimer (Khurshid et al., 2018). Trp234 is the only tryptophan residue in the Pgp3 sequence. Structural analysis observed the “hotspot” interaction between three Phe 6 residues of Pgp3n and one Trp234 of Pgp3c, which dominates the critical crystal packing interactions and is likely responsible for the immunogenic nature of the molecule (Galaleldeen et al., 2013).

To investigate the critical effect of amino residues on the pathogenesis of *Chlamydia*, we produced *C. muridarum* carrying an alanine (Ala) substitution of Tyr197 or Trp234. Both *in vitro* and *in vivo* experiments revealed that the Trp234 residue of Pgp3 played a significant role in *Chlamydia* infection and the induction of pathology.

## Materials and methods

### Chlamydial organisms and cell lines

*Chlamydia muridarum* organisms, including the plasmid-free clone (CMUT3) and intact plasmid transformants (pGFP), were kindly provided by Dr. Guangming Zhong at the University of Texas Health Science Center at San Antonio, USA. The new plasmid with a Pgp3 point mutation was generated from the intact pGFP::CM shuttle vector, which is described below. All the chlamydial organisms were propagated, purified, and stored as previously described (Zhong et al., 2001). The HeLa (human cervical epithelial carcinoma cells) cell line used in our current study was kindly provided by the Institute of Dermatology (PUMC, Nanjing, PRC). The cells were grown in 24-well plates, 12-well plates, and 6-well plates containing DMEM (Gibco, New York, USA) with 10% fetal bovine serum (FBS, Institute of Hematology, CAMS &PUMC, Tianjin, China) at 37°C in an incubator supplied with 5% CO_2_ and were infected with various chlamydial organisms.

### Construction of *C. muridarum* transformants with Pgp3 point mutations

To generate the Pgp3 point mutation, the pGFP::CM plasmid was amplified by appropriate primers that contain desired nucleotide substitutions (shown in Table 1), using AccuPrime pfx SuperMix (Life Technologies, Grand Island, NY). The PCR products were purified and digested with DpnI (BioLabs, Ipswich, MA) to remove the DNA template. The digested products were purified again and then transformed into *E. coli* XL-1 blue competent cells. Transformed plasmids were extracted from bacterial colonies positive for green fluorescence, and the extracted plasmids were partially sequenced for the validation of mutations. The plasmids with the desired mutation were transformed into *E. coli* ER2925 for amplification. The newly constructed plasmids were designated as pGFP::CMPgp3W234A and pGFP::CMPgp3Y197A. The two plasmids were introduced into *C. muridarum* plasmid-free organism (CMUT3) in the form of a purified elementary body, as previously reported (Gong et al., 2013). After transformation, the GFP-positive single cell was picked up and passaged for continuous five generations until the transformants with GFP were fully enriched. Then, the cell lysate was harvested, amplified, and plaque-purified as described before (Zhong et al., 2001). The stable transformants were named CMUT3-pGFP::CMPgp3W234A and CMUT3-pGFP::CMPgp3Y197A, shortened to Pgp3W234A and Pgp3Y197A below.

**Table 1:** Primers for Pgp3 gene mutagenesis.

	**Forward primer**	**Reverse primer**
Pgp3W234A	5′-gattagagagcggagttgtagcggttaatgctctatccaatgg-3′	5′-ccattggatagagcattaaccgctacaactccgctctctaatc-3′
Pgp3Y197A	5′-catgtgcgattagctatggcgcttcttctggtgtgcccaattt-3′	5′-aaattgggcacaccagaagaagcgccatagctaatcgcacatg-3′

### Immunofluorescence assay

HeLa cell monolayers grown on coverslips infected by *C. muridarum* transformants were fixed with 4% paraformaldehyde for 45 min at room temperature and permeabilized with 2% saponin for an additional 60 min. After blocking, the cell samples were incubated with mouse anti-Pgp3 or GlgA antibody, rabbit antichlamydial organism antibody, and Hochest (to mark DNA) overnight at 4°C. A goat anti-rabbit IgG secondary antibody conjugated with Alexa Fluor 488 and a goat anti-mouse IgG conjugated with Cy3 (Jackson ImmunoResearch, West Grove, PA) were used to incubate the cell samples at room temperature for 1 h. Immunofluorescence images were acquired using an immunofluorescence scanning microscope (Zeiss, Germany).

### Quantitative real-time PCR

The HeLa cells grown in 6-well plates infected by *C. muridarum* were harvested at 20 h post-infection using Trizol (Life Technologies, Grand Island, NY). The total RNA was extracted according to the protocol provided by the Direct-zol RNA MiniPrep Kit (Zymo Research, Irvine, CA). The extracted total RNA was prepared for cDNA with a ThermoScript reverse transcription-PCR (RT-PCR) system (Life Technologies, Grand Island, NY). The mRNA levels of genes were measured using TaqMan RT-PCR assays with iQ Supermix (Bio-Rad, Hercules, CA) and gene-specific primers (Table 2). The quantitative-PCR conditions are listed below: an initial denaturation step at 95°C for 3 min, amplification at 95°C for 15 s, and 60°C for 1 min for continuous 40 cycles. The mRNA levels of given genes were normalized to the copy number of *Chlamydia* IpdA mRNA in the corresponding samples.

**Table 2:** Q-PCR primers for plasmid encoded or regulated gene.

	**Forward primer**	**Reverse primer**
Pgp1	AAG AAG GGC GGC TTA TTC TG	AAG GCC GAG CTG CAA TTA T
Pgp2	CAC CAT CAA CAC GAA AGC ATA AA	CGC AGG CTT GAC TAC GAA ATA
Pgp3	GAT ACA CAA CCA TGT GCG ATT AG	GGG TGT CGA TCC GGA ATT AG
Pgp4	CCC GAG CTA GAC TTG AAA	CTC TTG TGG TAG AGT TCT AAG G
Pgp5	CTA TTC TCG GGC TGC AGA AA	GAT TAG TCG AAC TCC GGT CAT C
Pgp6	GCC TAT AAT GCG TTG GCT TAT	ATG CAG ACT TAT ACG GGA TTG A
Pgp7	GAA GAA ATG GGC GTG TGT TTA T	TCC GAA GCG CTG CTA TTT
Pgp8	AGA AGT TCA TGC ACG CTC TAA	GCG GGC AAT TTG TCT TAA CC
MOMP	AGA CCT TAC TAC AGC ACC TAC TC	AGC CAT GTA CGC AGC ATT AG
GlgA	GGA GAC GCA CTA TAC GGA CTA	CTC GTG GAG CGA AGT GAA TAA
TC0319	GTC ACA ACT GGA TGG CTA CTT	TCT GTT GGT TGG TTC CCA TAT T
TC0419	GAA CCT GTC CCT GCG AAT TA	AAG GCC CTA CTA GAG GAA GAA
TC0420	GAT AGG TCT ACG CAA GCC TAT AAT	CGA CTC TAC GTC TCT GCT ATT G
TC0421	GCT CCG ATT CTA TTC CGA CTT T	TCC TCC GAC CTC ATA GGT ATT T

### Mice infection and live organisms recovered from swabs or the oviduct/ovary

To increase mouse susceptibility for *C. muridarum* infection, each C3H/HeJ mouse was subcutaneously injected with 2.5 mg medroxyprogesterone (Depo-Provera; Pharmacia Upjohn, Kalamazoo, MI) 5 days prior to the infection. Then, the mice were inoculated with various *C. muridarum* transformants [2 × 10^5^ inclusion-forming units (IFUs)] intravaginally. To monitor the shedding from the lower genital tract (LGT) of mice, swabs from the vagina were acquired on days 3, 7, 14, 21, and 28 post-infection. Each swab was dissolved in 500 μl of ice-cold SPG, followed by vortexing with five glass beads for 1 min, and the *Chlamydia* organisms were released into the SPG supernatants. Then, the supernatants were titrated on HeLa cell monolayers in duplicate as described previously (Liu et al., 2014a). The total number of IFUs per swab was calculated based on the number of IFUs per view, the number of views per coverslip, dilution folds, inoculation doses, and the total sample volumes. The calculated total number of IFUs was presented as a Log10 scale.

To measure the live organism ascending into the upper genital tract (UGT), infected mice in parallel experiments were euthanized, and oviduct/ovary tissues were harvested on day 10 after infection. Oviduct/ovary tissues were immediately soaked in 250 μl ice-cold SPG buffer and then homogenized and sonicated, which released the live organisms to the SPG supernatant. After centrifugation at 3,000 rpm for 5 min to pellet large debris, the supernatants were titrated on HeLa cells as described above. The results are presented as log10 IFUs per tissue section homogenate. All the C3H/HeJ mice were purchased from The Jackson Lab (JAX)^®^, and the animal operation protocol was approved by the Ethics Committee of Tianjin Medical University General Hospital.

### Evaluating gross pathology and histopathology of the genital tract tissue of mice

On day 60 after infection, all the mice were euthanized, and total genital tracts, including the vagina, uterus, and oviduct/ovary, were harvested for evaluating gross pathology and histopathology. The genital tract tissues were isolated entirely and laid on a blue background for acquiring images. Hydrosalpinx on either side of the oviducts was determined to be hydrosalpinx positive. The severity of hydrosalpinx was scored according to the following criteria: (0) No hydrosalpinx; (1) Hydrosalpinx is only visible under a stereoscope; (2) Hydrosalpinx is visible with the naked eye but the size of hydrosalpinx is smaller than the homolateral ovary; (3) The size of the hydrosalpinx is equal to the homolateral ovary; and (4) The size of the hydrosalpinx is larger than the ovary. The scores from both sides are combined and regarded as the gross pathology score. The two independent researchers who evaluated the severity of hydrosalpinx were blind to the mice grouping. The incidence and the severity of hydrosalpinx in each group are statistically analyzed.

To monitor the histopathology, the genital tracts were fixed in 10% formaldehyde, embedded in paraffin, and serially sectioned longitudinally. The sections were examined using H&E staining, followed by observation under a microscope for evaluating the severity of inflammation. Two independent pathologists scored the severity of inflammatory cell infiltration according to the criteria: (0) No significant infiltration; (1) Infiltration at one single focus; (2) Infiltration at two to four foci; (3) Infiltration at more than four foci; and (4) Confluent infiltration. Images from each mouse were acquired under 10 × and 40 × objective lenses.

### Plaque size assay and growth curve evaluation

A plaque size assay was performed for evaluating *in vitro* growth properties of *C. muridarum*, following the protocol published previously (Huang et al., 2015). In brief, McCoy cell monolayers in 12-well plates were infected with extremely diluted chlamydial elementary bodies and centrifuged at 1,200 rpm for 1 h at room temperature. Then, the culture medium was changed into an overlay medium, containing 1 × Dulbecco's modified Eagle's medium, 10% fetal bovine serum, 1 μg/ml cycloheximide, and 0.55% of agarose. The infected McCoy cells were cultured in the overlay media for 5 days, followed by staining with 0.03% Neutral Red (Sigma) for 30 min at 37°C. Images were acquired with an Epson scanner, and the diameters of plaques were measured with the custom MATLAB program plaque detector (http://www.mathworks.com/matlabcentral/fileexchange/48860-plaque-detector).

A growth curve assay was performed to evaluate *C. muridarum in vitro* proliferation in a single generation. All *C. muridarum* transformants were inoculated onto McCoy monolayers grown in 24-well plates at an MOI of 0.5 (to avoid over infection), with the assistance of DEAE pretreatment and centrifugation. At different time points post-infection, cell lysates were harvested from each culture and a number of live organisms were titrated (in the form of the elementary body) on the HeLa cell monolayers.

### Statistical analysis

All the IFU data presented in the form of Log10 were analyzed using the Kruskal–Wallis test. Semi-quantitative data including the pathology scores were analyzed using the Mann–Whitney U rank-sum test, while the incidences of hydrosalpinx were analyzed using Fisher's exact test. Quantitative data including diameters of plaque size were analyzed using Student's *t*-test. All the statistical analyses mentioned above were performed using IBM SPSS 22.0 software.

## Results

### Generation and characterization of *C. muridarum* with a Pgp3 point mutation

**Figure 1:**
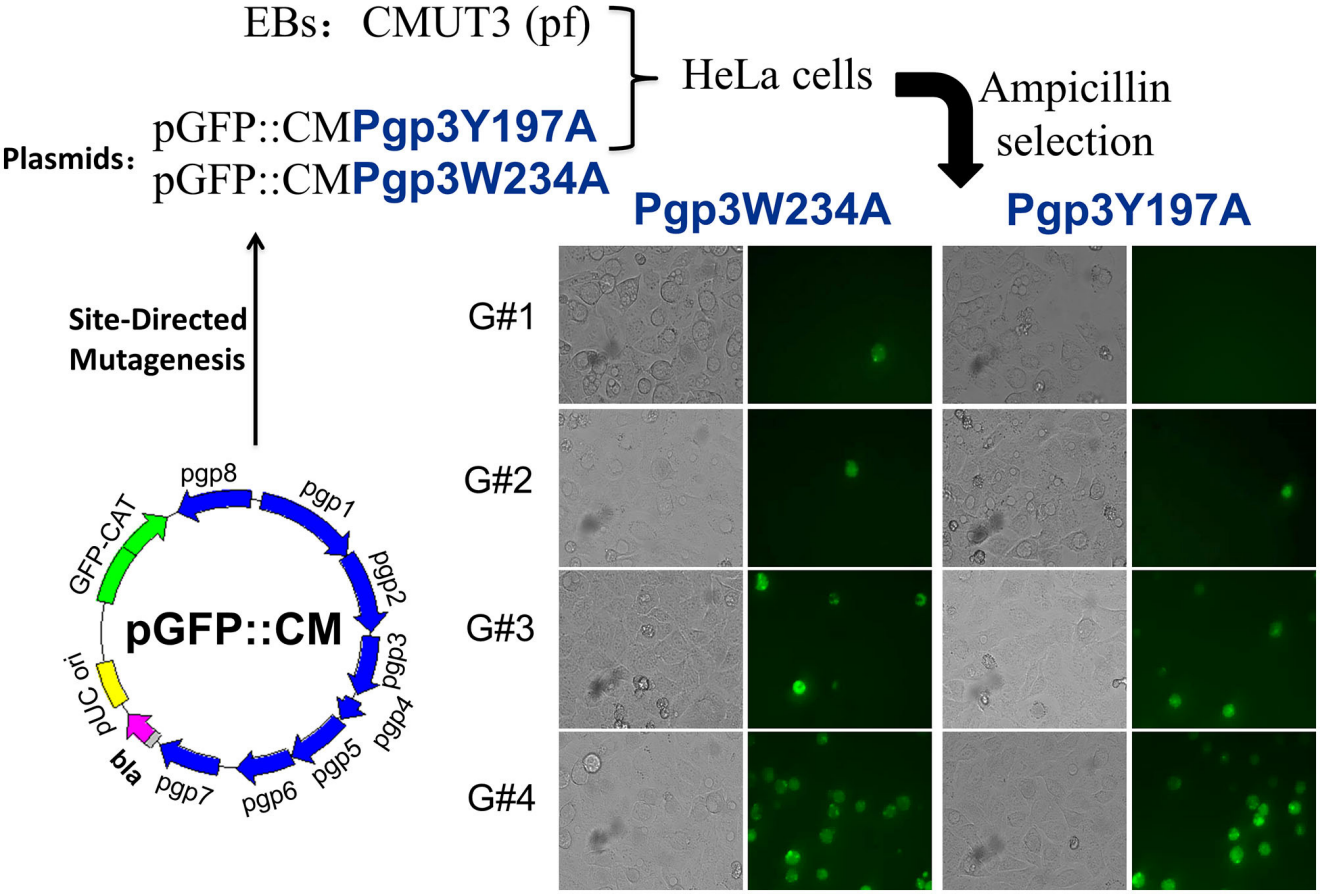
Construction of Pgp3 point mutants. Site-directed mutagenesis was performed in the intact plasmid pGFP::CM to generate pGFP::CMPgp3Y197A and pGFP::CMPgp3W234A, which have an alanine substitution at Pgp3 Tyr197 or Trp234, respectively. The mutated plasmids were transformed into a plasmid-free clone CMUT3 and selected with ampicillin. After GFP-positive inclusion body pick-up selection for continuous five generations, the stable transformants Pgp3Y197A and Pgp3W234A were obtained.

We generated pGFP::CM plasmids with alanine substitutions at positions 234 (tryptophan) or 197 (tyrosine) of Pgp3 *via* site-directed mutagenesis. Then, the two mutated plasmids were transformed into a *C. muridarum* plasmid-free clone (CMUT3), cultured in a HeLa cell monolayer for 12 h without ampicillin, and subsequently cultured with ampicillin for 20 h. These cells were continuously passaged and selected with ampicillin for four generations, and then, GFP-positive inclusions were enriched. After selecting single clones using a plaque assay, CMUT3 transformants harboring plasmid mutants were named Pgp3W234A or Pgp3Y197A (Figure 1).

**Figure 2:**
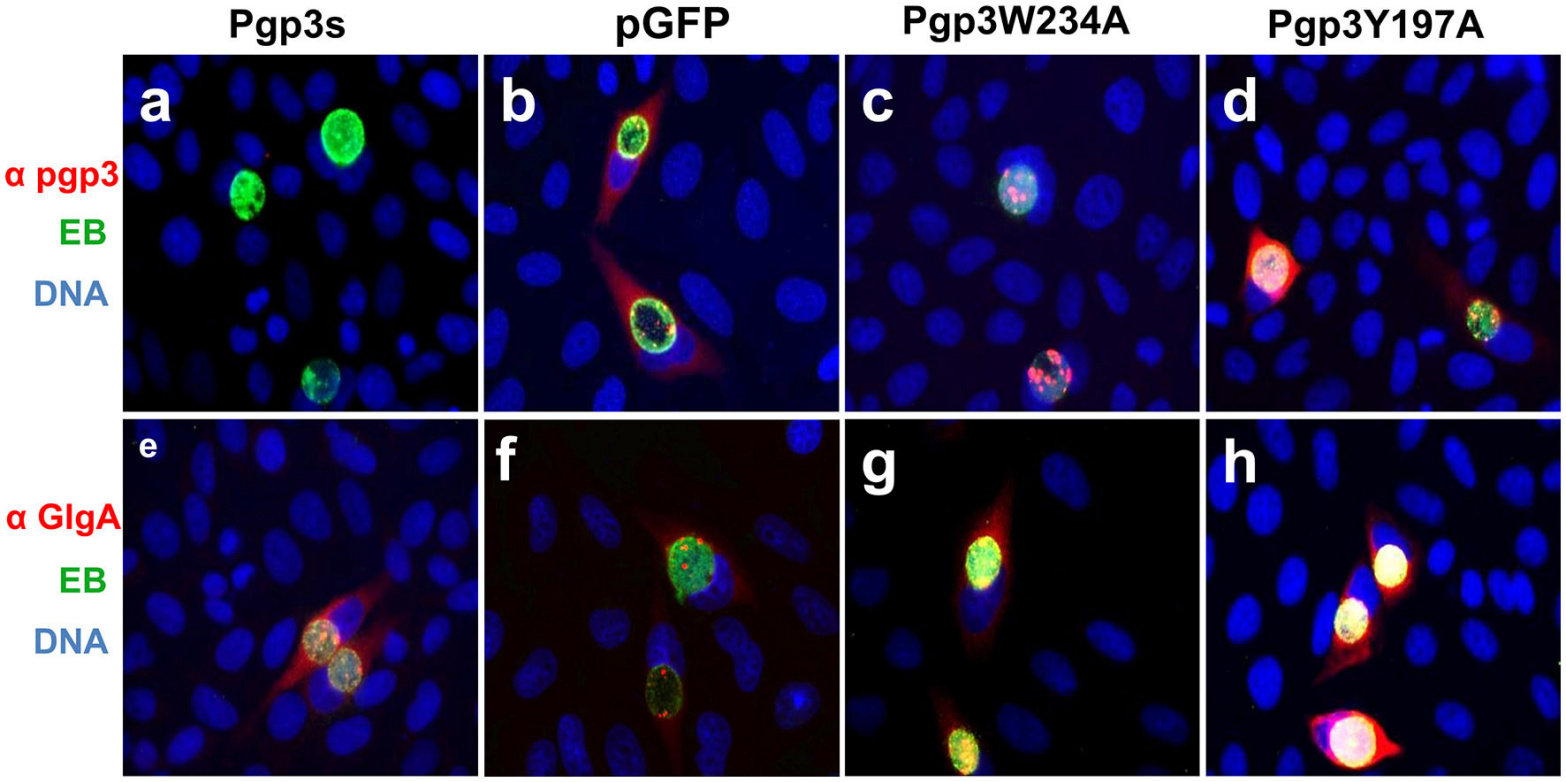
Effect of Pgp3 point mutation on Pgp3 and GlgA protein expression. The newly constructed Pgp3 point mutations (Pgp3W234A and Pgp3Y197A), the organism with a premature termination codon inserted into the Pgp3 9th residue (named as Pgp3s), and *Chlamydia muridarum* organism with intact plasmid pGFP (named as pGFP) were used to infect HeLa cells. The infected cells were assessed using immunofluorescence staining for Pgp3 (red, **a–d**) or GlgA (red, **e–h**) proteins, chlamydia (green), and DNA (blue). Notably, the mutated Pgp3W234A protein was mostly localized to the inclusion body and had less secretion into the cytosol.

We further assessed Pgp3 and GlgA protein expression in the two transformants. CMUT3 containing an intact pGFP::CM plasmid was used as a positive control, while the transformants introduced with a premature stop codon in the Pgp3 gene (Pgp3s) were considered a negative control. As expected, neither of the two mutants showed defective GlgA expression (Figure 2). However, although Pgp3 immunoactivity was detected in cultures infected with Pgp3Y197A or Pgp3W234A *C. muridarum*, the Pgp3 protein in Pgp3W234A was detected only in inclusion bodies of *Chlamydia*, not in the host cell cytoplasm. This suggests that an alanine substitution in Trp234 may impair the secretion of Pgp3 into the host cell cytoplasm.

**Figure 3:**
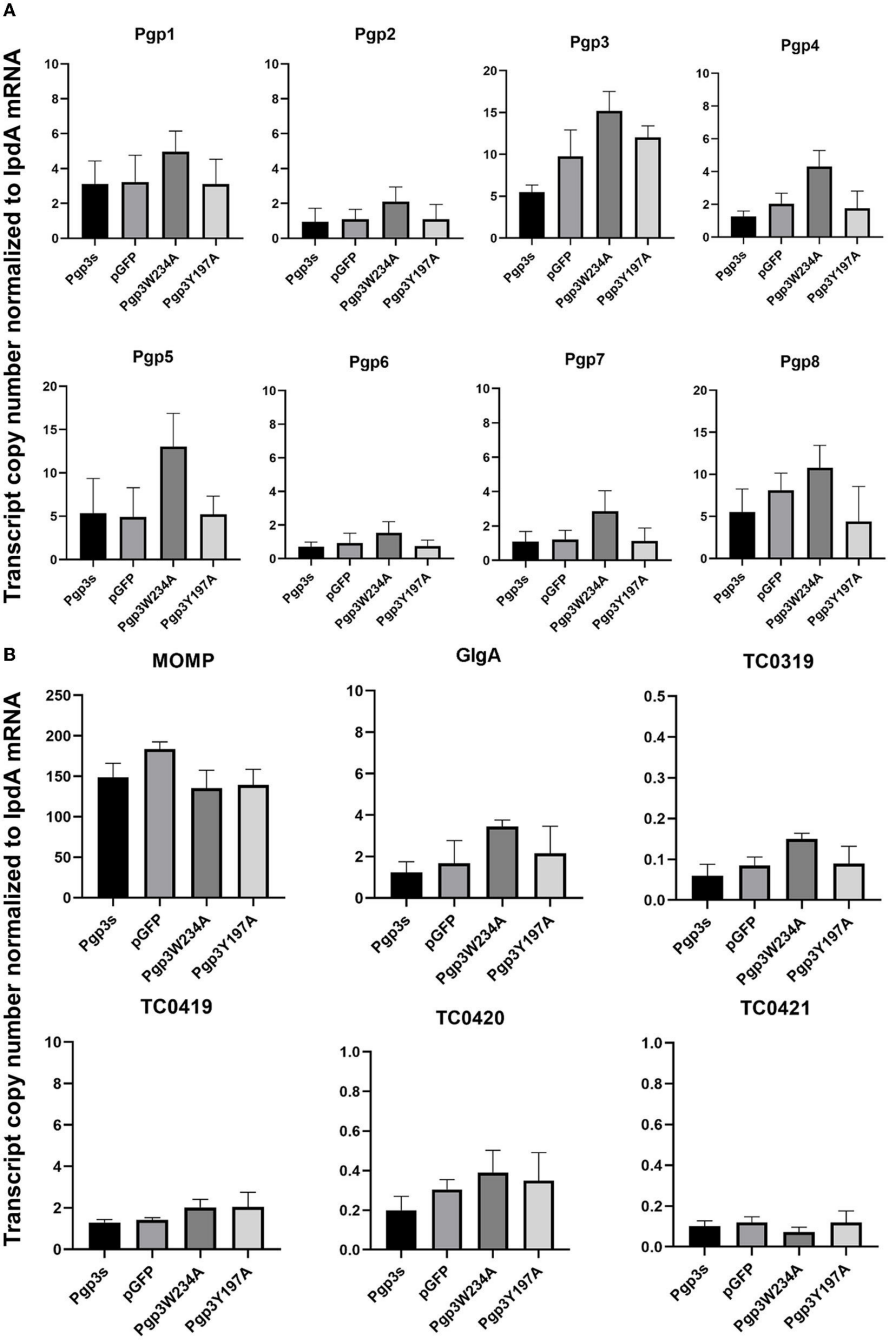
Effect of Pgp3 point mutation on the mRNA levels of plasmid-encoded gene and plasmid-regulated genes. The organisms indicated along the X-axis were used to infect HeLa cells. The cell culture was harvested at 20 h post-infection and RNA was extracted for RT-qPCR quantitation of eight plasmid-encoded genes (**A**: Pgp1–Pgp8) and six plasmid-regulated genomic genes (**B**: momp, GlgA, TC3019, and TC4019-4021). The transcript copy number of 14 genes normalized to IpdA is shown along the Y-axis.

Next, we evaluated the effects of *C. muridarum* Pgp3 point mutations on the mRNA levels of plasmid-encoding genes. The result showed that there were no significant differences between the Pgp3W234A mutant and intact control (Figure 3A). To further investigate whether Pgp3 point mutations impact the transcriptional levels of chromosomal genes, we assessed five plasmid-dependent chromosomal genes; all of them showed a more than 3-fold change in transcriptional level between plasmid-free and intact *C. muridarum* organisms (Liu et al., 2014b). In addition, neither of the cultures infected with Pgp3W234A or Pgp3Y197A displayed reduced mRNA levels of the plasmid-dependent genes GlgA, TC0319, TC0419, TC0420, and TC0421. Thus, we deduce that both Pgp3 point mutations have no impact on the expression of plasmid-encoding genes and plasmid-dependent chromosome genes.

### Pgp3W234A mutant does not induce severe hydrosalpinx in C3H/HeJ mice after intravaginal infection

**Figure 4:**
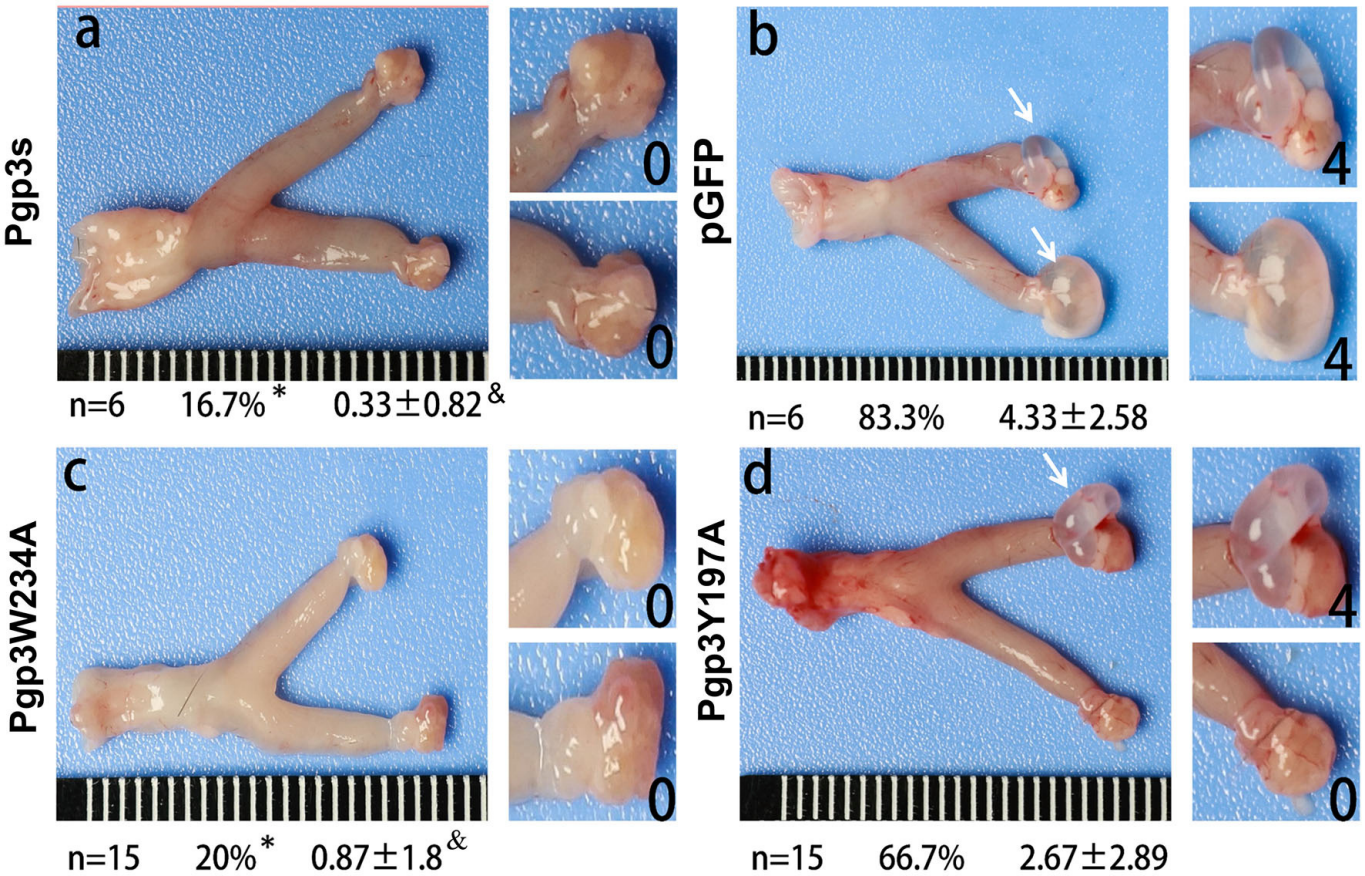
*Chlamydia muridarum* with Pgp3 Trp234 mutation failed to induce severe hydrosalpinx in C3H/HeJ mice after intravaginal infection. CMUT3 transformants [Pgp3s, **a**; pGFP (intact), **b**; Pgp3W234A, **c**; and Pgp3Y197A, **d**] were used to infect C3H/HeJ mice intravaginally. On the 60th day post-inoculation, the genital tracts were harvested. The gross pathology in the upper genital tract (UGT) was recorded and scored. Representative images of the genital tract **(left)** and oviduct/ovary **(right)** are shown. The total number of mice (*n*), the incidence (%), and the severity score of hydrosalpinx (mean ± SD) are listed below each image (*P < 0.05, Fisher's exact test; ^&^*P* < 0.05, Mann–Whitney U rank-sum test).

*C. muridarum* transformant without Pgp3 protein expression (Pgp3s) failed to induce hydrosalpinx in C3H/HeJ mice after intravaginal infection. Analyses of the structure of the Pgp3 protein indicated that Trp234 is essential for interactions between neighboring C-terminal domains of Pgp3 molecules, while Tyr197 is involved at K+ cation-binding sites (Khurshid et al., 2018). To identify the critical amino acid of the Pgp3 protein that induces pathology, we used the Pgp3W234A and Pgp3Y197A mutants to infect C3H/HeJ mice intravaginally. After 60 days of infection, mice were euthanized, and their genital tracts were harvested to assess oviduct pathology. As shown in Figure 4, both pGFP and Pgp3Y197A induced severe oviduct hydrosalpinx. In contrast, only 20% of the C3H/HeJ mice infected with Pgp3W234A developed hydrosalpinx (severity score 0.87 ± 1.8) as compared with that induced by Pgp3s (incidence: 16.7%, severity score 0.33 ± 0.82). This indicates that Trp234 is essential for *C. muridarum* to induce oviduct pathology.

### Effect of Pgp3 point mutations on *C. muridarum in vitro* growth and survival in the lower genital tracts of mice

**Figure 5:**
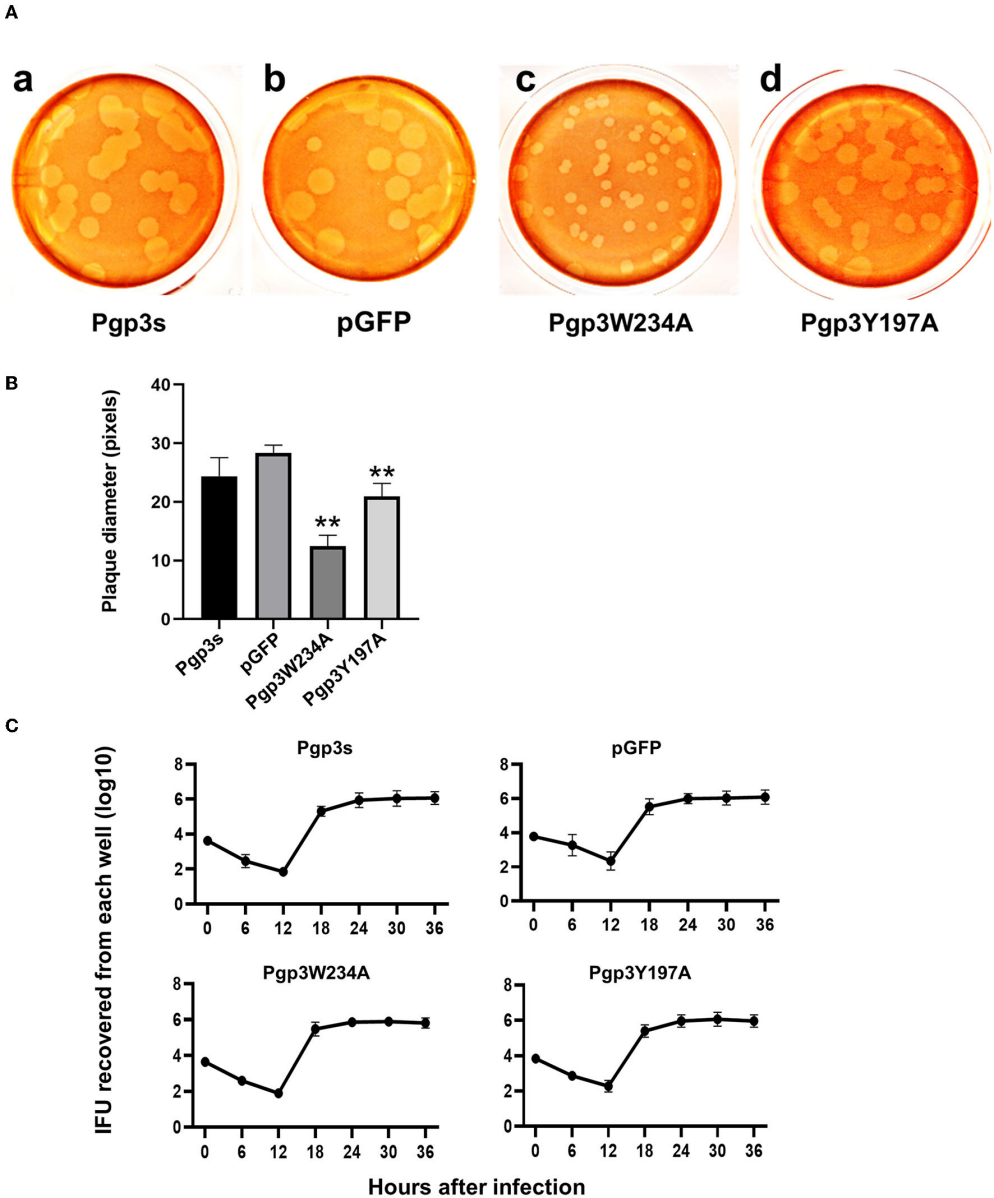
Comparison of growth *in vitro* among *C. muridarum* transformants. **(A)** A plaque size assay was performed for the organism Pgp3s (a), pGFP (intact, b), Pgp3W234A (c), and Pgp3Y197A (d). McCoy cell monolayers infected by the above organisms were stained with neutral red after inoculation in a 12-well plate for 5 days. **(B)** Plaque sizes shown in **(A)** were quantified in pixels using plaqueDetector (**P < 0.01; Student's *t*-test). **(C)** Comparison of primary growth curves among various transformants. The McCoy cell monolayers infected with various transformants were harvested at 6, 12, 18, 24, 30, and 36 h post-infection for titrating the number of live elementary bodies on fresh monolayers of McCoy cells. The number of elementary bodies from each culture at the indicated time points was calculated as IFU per culture, as listed along the Y-axis in a Log scale.

Previous studies have demonstrated that Pgp3 plays a critical role in promoting *C. muridarum* survival in both the lower and upper genital tracts, the latter of which is considered essential to cause oviduct pathology (Liu et al., 2014a). To investigate whether the Pgp3W234A mutant impairs *Chlamydia* survival, a plaque size assay was performed to assess *in vitro* growth defects. As expected, Pgp3W234A produced significantly smaller plaques than the pGFP intact control, while Pgp3Y197A produced intermediate-sized plaques (Figures 5A, B). Surprisingly, the plaque sizes of Pgp3s were similar to those of pGFP control, consistent with a previous report (Huang et al., 2015). It is worth mentioning that although a premature stop codon in the pgp3 gene (Pgp3s) produced comparable plaque sizes to intact controls, the in-frame deletion of Pgp3 led to dramatically smaller plaques than those formed by intact *C. muridarum* (Huang et al., 2019).

Because the plaque size assay reflects cumulative results of multiple generations of *Chlamydia* inclusion bursts, we further evaluated the one-step growth curves among these organisms. After infecting McCoy monolayers via DEAE pretreatment and centrifugation, live organisms were recovered (Figure 5C). All of the organisms displayed similar growth kinetics during the entire infection course. No significant differences in terms of live organism yields were found at any time point. This suggests that all of the organisms in our study shared similar intracellular growth abilities in one generation. The smaller plaques formed by Pgp3W234A and Pgp3Y197A (Figure 5A) were probably due to the reduced natural infectivity and spreading of live organisms.

**Figure 6:**
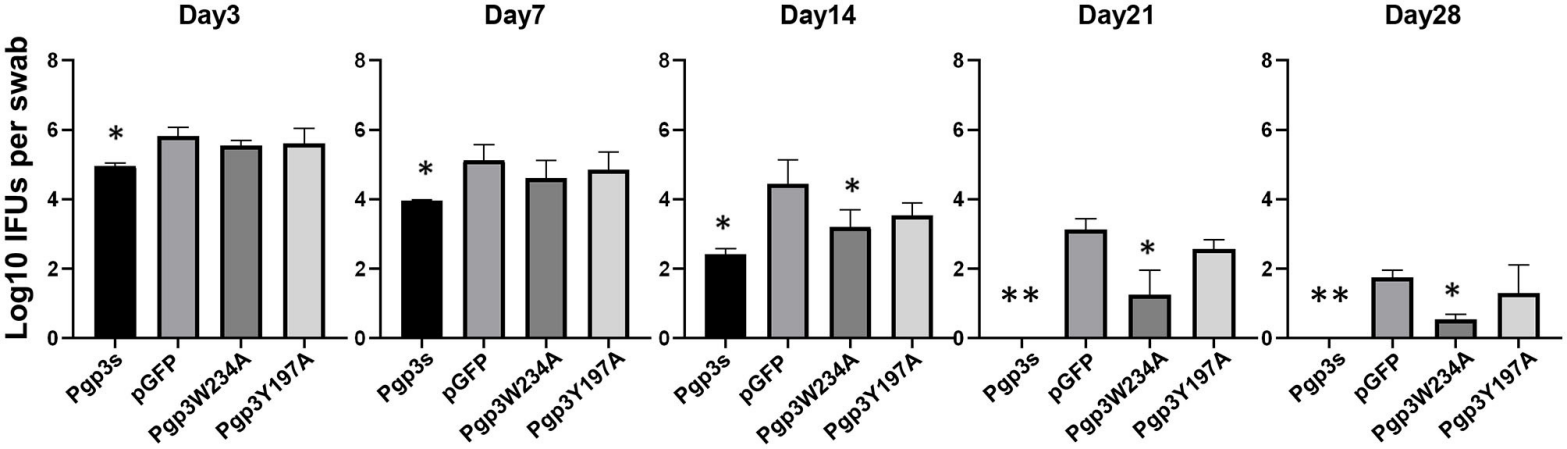
Effect of Pgp3 point mutation on live *C. muridarum* organism shedding from the mouse LGT. C3H/HeJ mice were intravaginally infected with *C. muridarum*, listed along the X-axis. On the indicated days post-infection, vaginal swabs were obtained, and live organisms were titrated by infecting HeLa cell monolayers. The amounts of live organisms are presented as log10 IFUs. Mice infected with Pgp3W234A or Pgp3s shed reduced live organisms compared with the intact organism pGFP. *P < 0.05, **P < 0.01; Kruskal–Wallis test.

Because the two mutants showed defective growth *in vitro*, we evaluated the effect of point mutations on the infection course of *C. muridarum* in the lower genital tracts of the mice. Live organisms shed from the lower genital tracts were monitored on days 3, 7, 14, 21, and 28 after intravaginal inoculation. The overall levels of shedding were reduced in mice with Pgp3s. The lengths of the infection course were comparable among the other three organisms. However, mice infected with Pgp3W234A showed decreased IFU shedding on days 14, 21, and 28, suggesting that the alanine substitution of Pgp3 Trp234 impaired *Chlamydia* survival (Figure 6).

### Effect of Pgp3 point mutations on *C. muridarum* ascending to the mice oviduct

**Figure 7:**
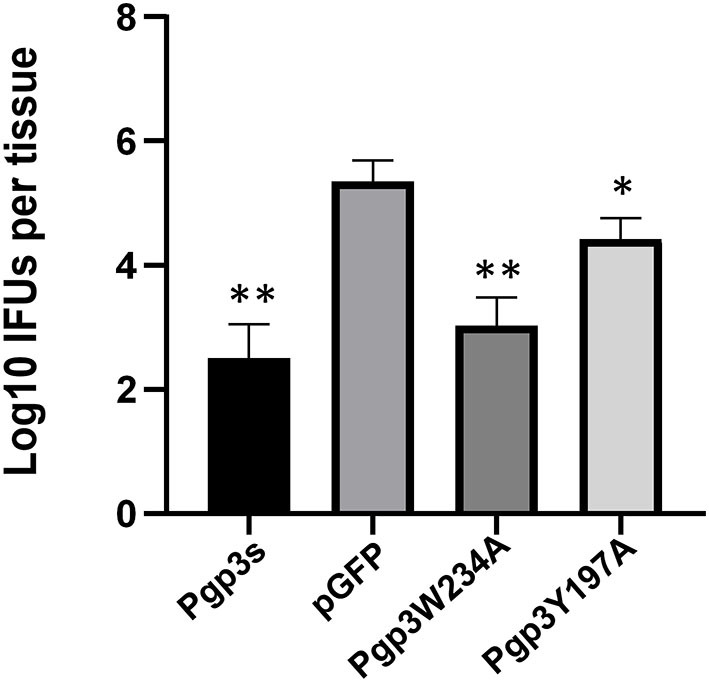
Live organisms recovered from mice oviduct/ovary tissue following infection with various *C. muridarum* transformants. C3H/HeJ mice intravaginally infected with various transformants described in the legend of Figure 4 were euthanized on day 10 after infection. The oviduct/ovary tissue from the upper genital tract was homogenized for titrating live organisms. Notably, the number of live organisms recovered from the oviduct/ovary tissue of mice infected with Pgp3s, Pgp3W234A, or Pgp3Y197A was significantly reduced compared with that from the intact pGFP group. *P < 0.05, **P < 0.01; Kruskal–Wallis test.

Since an adequate number of live organisms must ascend into the oviduct to induce hydrosalpinx, we further assessed the effect of two-point mutations on *C. muridarum* ascension. On day 10 after intravaginal infection, oviduct/ovary tissue was acquired from mice. The number of live organisms recovered from oviduct/ovary tissue was significantly lower in Pgp3s infected mice than in pGFP infected mice, which confirms a previous report (Liu et al., 2014a). Notably, the number of live organisms recovered from oviduct/ovary tissue in mice infected with Pgp3W234A phenocopied that in mice infected with Pgp3s, while the mice infected with Pgp3Y197A presented intermediate phenotypes (Figure 7). This indicates that both point mutations of Pgp3 impacted *C. muridarum* ascension and survival in the oviduct/ovary tissue. However, Pgp3W234A had the greatest effect.

### Pgp3W234A *C. muridarum* is less inflammatory in the mouse oviduct

**Figure 8:**
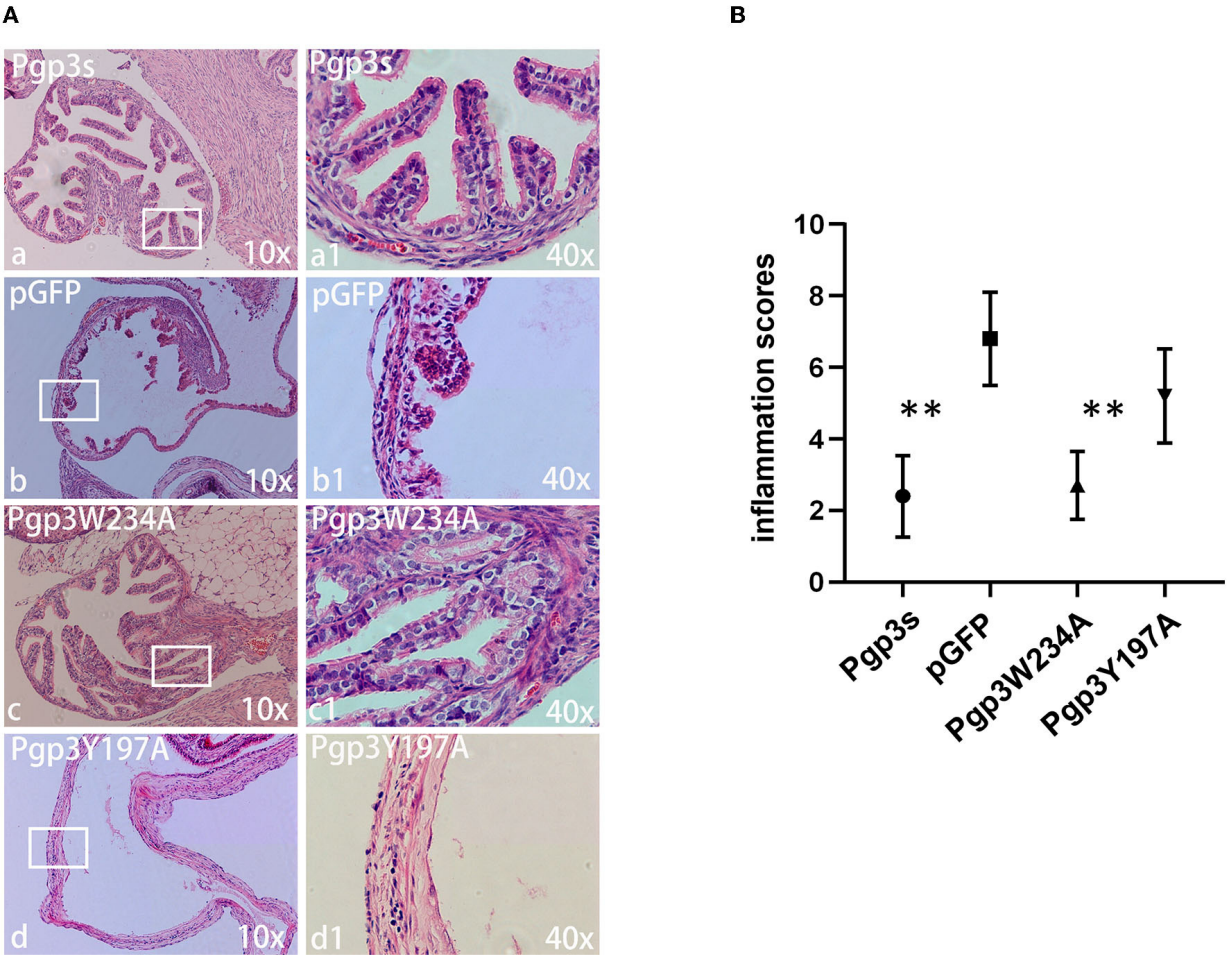
*Chlamydia muridarum* with Pgp3 Trp234 mutation failed to cause severe inflammatory cell infiltration in C3H/HeJ mice oviducts after intravaginal inoculation. **(A)** The genital tract tissues described in Figure 4 were subjected to histopathology examination, evaluating oviduct dilation and inflammatory cell infiltration. Representative images from each group at 10× magnification (a–d) and 40× magnification (a1–d1, areas demarcated by white rectangles) are shown. **(B)** The severity of inflammatory cell infiltration was semi-quantified. Notably, organisms with mutation in Pgp3Y197, but not in Pgp3W234, induced severe oviduct dilation inflammatory cell infiltration. **P < 0.01; Mann–Whitney U rank-sum test.

Another essential factor for hydrosalpinx is a robust immune response. Thus, we monitored oviduct dilation and inflammatory cell infiltration. On day 60 after intravaginal infection, mice were euthanized, and their whole genital tracts were obtained for histological sectioning. As expected, Pgp3s induced mild inflammatory cell infiltration (inflammation score: 2.4 ± 1.14) and luminal dilation. Interestingly, Pgp3W234A failed to develop significant inflammatory cell infiltration and oviduct dilation, whereas Pgp3Y197A did (Figure 8). These results confirm the gross pathology findings described above (Figure 4). According to the inflammatory score, it is clear that the Pgp3W234A mutant failed to induce a robust immune response after ascending into oviduct tissue.

## Discussion

In a previous study, we identified Pgp3 as an important virulence factor for oviduct pathogenesis caused by *C. muridarum* and found that each of its domains was essential for virulence (Liu et al., 2014a; Huang et al., 2019). The X-ray crystal structure of Pgp3 revealed two critical amino residues located at the C-terminal domain: Tyr197 as a key component of a predominant cation-binding site and Trp234, which interacts with Phe 6 and forms an NTD-to-CTD arrangement (Khurshid et al., 2018). In this study, we constructed *C. muridarum* strain transformants carrying plasmids with a Pgp3 point mutation on each of two amino residues. Further *in vitro* characterization revealed that although neither of the two mutants affects plasmid-encoded genes or plasmid-related chromosomal genes at the transcriptional level, the Pgp3 protein produced by the Pgp3W234A mutant presented impaired secretion into the host cell cytosol. More importantly, the mutant lost the ability to induce severe oviduct pathology, possibly due to reduced survival, reduced ascension to the oviduct, and attenuated induction of a robust inflammatory response in the oviduct tissue.

Accumulated evidence has proven Pgp3 to be a virulence and *in vivo* fitness factor to support chlamydial infection and that it is also involved in stimulating host immune responses (Gong et al., 2013; Liu et al., 2014a). It improves the fitness of CM in the gastrointestinal tract by enhancing resistance to gastric acid and overcoming a CD4+ T-cell barrier in the small intestine (Huo et al., 2020; Zhong, 2021). In a recent study, a Pgp3-deficient mutant was more susceptible to death due to lactic acid exposure in the genital tract, suggesting the possible mechanisms by which Pgp3 promotes *Chlamydia* survival after infection (Zhang et al., 2019). During infection, *Chlamydia*-infected epithelial cells become inflammatory and surrounded by neutrophils. Mucosal effectors such as human antimicrobial peptide LL-37 are secreted by neutrophils and epithelial cells, which may suppress *Chlamydia* survival and ascension. *Chlamydia* may use Pgp3 to block detrimental inflammation and promote survival (Hou et al., 2019). Further investigation is required to test whether Trp234 plays a role in this process.

Oviduct inflammation is needed to induce hydrosalpinx. In fact, *Chlamydia* organisms activate many inflammatory pathways to promote oviduct inflammation. Purified Pgp3 protein stimulates macrophages to release inflammatory cytokines (Li et al., 2008). It induces the expression of a series of host inflammatory cytokine genes, including interleukin-6 (IL-6), IL-8, tumor necrosis factor alpha-induced protein 3 (TNFAIP3), and chemokine C-X-C motif ligand 1 (CXCL1), after stimulating HeLa cells (Cheong et al., 2023). The involvement of tumor necrosis factor receptor 1 (TNFR1) in *Chlamydia* pathogenesis is supported by crystal analyses that have shown that the Pgp3 C-terminal trimerization domain resembles the TNF-like domain (Galaleldeen et al., 2013). However, there is still no direct evidence to support the suggestion that Pgp3 promotion of tubal inflammation requires the TNFR1 pathway. Persistent and subverted immune responses may inevitably contribute to the development of inflammatory sequelae such as tubal fibrosis. In addition, it is important to note that monomeric Pgp3 fails to trigger protective immunity against *Chlamydia*, indicating that the homotrimer conformation greatly contributes to the host immune response (Peng et al., 2022). Further exploration is required to test whether the Pgp3W234A mutant impacts the natural trimeric conformation of Pgp3, thus altering the induction of the immune response of hosts.

## Data availability statement

The raw data supporting the conclusions of this article will be made available by the authors, without undue reservation.

## Ethics statement

The animal study was reviewed and approved by the Ethics Committee of Tianjin Medical University General Hospital.

## Author contributions

YL, YH, and YS conceived and designed the experiments. HW and YH performed the experiments. HW and YS analyzed the data. YL and YH wrote the paper. All authors contributed to the article and approved the submitted version.
